# The Effectiveness of *Varroa destructor* Infestation Classification Using an E-Nose Depending on the Time of Day

**DOI:** 10.3390/s20092532

**Published:** 2020-04-29

**Authors:** Andrzej Szczurek, Monika Maciejewska, Żaneta Zajiczek, Beata Bąk, Jakub Wilk, Jerzy Wilde, Maciej Siuda

**Affiliations:** 1 Faculty of Environmental Engineering, Wroclaw University of Science and Technology, Wybrzeże Wyspiańskiego 27, 50-370 Wrocław, Poland; andrzej.szczurek@pwr.edu.pl (A.S.); zaneta.zajiczek@pwr.edu.pl (Ż.Z.); 2 Apiculture Department, Warmia and Mazury University in Olsztyn, Sloneczna 48, 10-957 Olsztyn, Poland; beata.bak@uwm.edu.pl (B.B.); teofil.wilk@uwm.edu.pl (J.W.); jerzy.wilde@uwm.edu.pl (J.W.); maciej.siuda@uwm.edu.pl (M.S.)

**Keywords:** gas sensor, varroosis, honeybee, disease, detection, indoor air

## Abstract

Honey bees are subject to a number of stressors. In recent years, there has been a worldwide decline in the population of these insects. Losses raise a serious concern, because bees have an indispensable role in the food supply of humankind. This work is focused on the method of assessment of honey bee colony infestation by *Varroa destructor*. The approach allows to detect several categories of infestation: “Low”, “Medium” and “High”. The method of detection consists of two components: (1) the measurements of beehive air using a gas sensor array and (2) classification, which is based on the measurement data. In this work, we indicate the sensitivity of the bee colony infestation assessment to the timing of measurement data collection. It was observed that the semiconductor gas sensor responses to the atmosphere of a defined beehive, collected during 24 h, displayed temporal variation. We demonstrated that the success rate of the bee colony infestation assessment also altered depending on the time of day when the gas sensor array measurement was done. Moreover, it was found that different times of day were the most favorable to detect the particular infestation category. This result could indicate that the representation of the disease in the beehive air may be confounded during the day, due to some interferences. More studies are needed to explain this fact and determine the best measurement periods. The problem addressed in this work is very important for scheduling the beekeeping practices aimed at *Varroa destructor* infestation assessment, using the proposed method.

## 1. Introduction

The Western honey bee (*Apis mellifera*) plays a key role in a range of human activities, including nutrition, medicine and agriculture. These insects are among the most important pollinators of plants. They pollinate wildflowers and many crops; thus, our food sources depend on bees [1,2]. In recent years, all over the world, there has been a strong decrease in the number of *Apis mellifera* colonies [3,4,5]. This phenomenon is caused by multiple factors, including the use of pesticides in agriculture, the presence of pollutants in the environment, mite infections, fungal diseases, viruses, climate change, malnutrition and starvation linked to environmental degradation [6]. Honey bees as living organisms are subject to a number of pests, infections, diseases and disorders. The spread of diseases or parasites is fast, because honey bees are social insects that live in colonies. Currently, honey bee decline is a serious threat to global agricultural security and productivity [7].

A bee colony has its defense mechanisms; however, sometimes they are too weak. In this situation, insects need intervention from a beekeeper. In apiculture, one of the crucial issues is the inspection of honey bee colonies for their health [8]. Beekeepers should be able to recognize bee diseases and parasites and to differentiate the levels of infestation. The traditional assessment of the health of a honey bee colony is based on manually inspecting the hives and visual observations of flight activity [9,10,11]. This strategy is strongly subjective, expensive, time consuming and requires great experience. It is almost impossible for human eyes to identify the characteristic symptoms of honey bee diseases immediately. Successful manual inspection requires long periods of observation. This leads to delays in the prevention and treatment of infection. Sometimes, a specific expertise is required, in order to ensure reliability. Hence, instrumental methods using measuring devices are recommended for the future of honey bee health assessment.

A variety of measurement equipment has been proposed for the extended monitoring of honey bee colonies [12,13,14,15,16]. Usually, these are based on the measurements of temperature, air humidity, sound, vibrations of the hive, the counting of outgoing and incoming bees, video observations and weighing. The chemical composition of beehive air is relatively rarely taken into account as a source of information about honey bee diseases. This is due to several reasons, e.g., the air inside beehives consists of many volatile compounds, their concentrations are relatively low, and there are not established chemical indicators of honey bee diseases. Additionally, instruments for the qualitative and quantitative analysis of complex gaseous mixtures are complicated and expensive [17]. 

The cost of traditional analytical instruments, such as infrared analyzers, Fourier transform infrared spectrometers (FT-IR spectrometers) or gas chromatographs with the appropriate detectors (usually flame ionization (FID), a tuned mass spectrometer (MS) or other mass-selective detectors), is prohibitive. They are beyond the reach of the average private user. Most of this equipment does not have the detection limit required for trace-level determinations. The measurements are time-consuming, and trained and experienced personnel is required. The instruments are very often bulky in size, heavy in weight, inconvenient to transport and require a high consumption of energy. The limitations of traditional analytical instruments directed our focus to a measurement apparatus described as an electronic nose (E-nose) [18,19,20]. In this work, we propose the use of E-nose technology for the detection of varroosis.

Varroosis is one of the most important honey bee diseases [21,22,23,24]. It is caused by *Varroa destructor* (*V.d.*)—a small, external parasite of adults and brood. This mite is a highly destructive pest that can severely reduce honey production, cause malformation of bee’s legs, wings, body segments and induce high mortality during winter. *V.d.* infestations often leave honey bee colonies weakened and more susceptible to disease. Mites are spread to other colonies through drifting and robbing. Their high reproductive potential makes managing these mites a considerable challenge for beekeepers [25,26,27,28].

The most widely accepted definition of the E-nose was published by J.W. Gardner and P.N. Bartlett [29,30,31]. This states: “An electronic nose is an instrument which comprises an array of electronic chemical sensors with partial specificity and an appropriate pattern recognition system capable of recognizing simple or complex odours”. These devices are not dedicated to the qualitative and quantitative analysis of complex mixtures. The properties of gas sensors mean that E-noses are mainly preferred for the classification of complex gaseous mixtures [32,33,34,35]. 

The concept of the E-nose, as a measuring device for the detection of varroosis, is based on the assumption that *V. d.* infestations influence the chemical composition of the air inside a bee hive. It is extremely difficult to determine the changes of particular components of the beehive air in a simple way. Hence, we propose an approach based on the classification of air quality. In our opinion, this idea allows for the detection of the infestation rate of the honey bee colony. The results of our previous studies of this issue support this assumption [36,37,38,39].

An E-nose dedicated for the detection of varroosis can be used as a fixed, stationary, automated instrument or a portable, direct-reading device. The first type of equipment is appropriate for continuous measurements, which normally cover the duration of one measurement program without any interruption. The stationary equipment can operate over extended periods (weeks or months) with minimal operator intervention. Continuous fixed-point monitoring is recommended in situations where the gas presents strong variability in the chemical composition. The time series analysis allows for the reduction of the influence of measurement signal fluctuations on the detection process. However, semiconductor gas sensors seldom reach an equilibrium state with tested gas [40]. This is due to their slow dynamics and the rapid fluctuations of the gas compositions. 

The monitoring based on a stationary E-noses is expensive, as one instrument is dedicated for only one beehive. In addition, the measurement equipment is under the influence of meteorological conditions [36,37,38,39]. Metrological problems result also from the long exposure of sensors to volatile compounds, which are components of the gas permanently flowing through the measurement chamber. The adsorbed gases can be a source of serious errors, as the continuous sampling does not allow for cleaning operations of the measurement system. The continuous mode of operation also requires frequent calibration. Monitoring based on fixed-location instruments is not suitable for large-scale inspections.

For large-scale survey work, portable instruments are generally more appropriate. These devices are mainly dedicated for periodic measurements, which are accomplished in various places. Thereby, the detection of varroosis in many honey bee colonies is possible with such an instrument. In practice, inspections at several locations are needed more than single-point measurements. The monitoring using discontinuous measurements allows for a mode of operation based on the principle of “exposure cleaning”. This method ensures the rapid exchange of gas inside the measurement chamber and the regeneration of sensors. Hence, the memory effect can be significantly reduced. The employment of automatic sampling in direct-reading instruments allows continuous and uninterrupted measurements to be realized. Therefore, monitoring based on portable devices usually consists of a series of measurement sessions, which are realized according to a monitoring plan. They should be relatively short and provide representative data.

The chemical composition of the air inside a beehive presents strong temporal variability [14,41,42,43,44,45,46,47,48,49,50,51,52]. For this reason, the key issue for the successful detection of the bee colony disease may be the time of day when individual measurement sessions are performed. Otherwise, there is a certain probability of obtaining unrepresentative gas samples. The factors influencing the measurement results and classification process very often present specific patterns of occurrence. Diurnal patterns of variation of beehive air composition are likely to exist [41,42,43,44,45,46,47]. Thus, the information about the specific periods of day can be useful in the planning of a measurement program. This is a prerequisite of collecting the representative data. The appropriate time of measurements allows for better effectiveness of the detection. Additionally, it is important from an economical point of view, as beekeepers need to spend a certain amount of time, labor and money to perform the measurements. The aim of this work was to examine the dependence between the effectiveness of *V.d.* infestation classification and the time of day, when the measurements are performed.

## 2. Materials and Methods

### 2.1. Bee Colonies and Their V.d. Infestation Rates

Fifteen honey bee colonies were included in this study. The colonies were examined with respect to the *V.d.* infestation rate using a flotation method. This is an established manual approach of the infestation rate assessment [9,53]. It consists of (1) collecting bees from the colony, preferably from honeycombs with brood; (2) anesthesia; (3) placing the bees in a jar with a mixture of water and detergent or alcohol and shaking; (4) pouring the contents of the container over a first sieve (aperture: 3–4 mm) to collect all the bees; (5) flushing the sieves with a large amount of water; (6) collecting mites on a second sieve (aperture < 0.5 mm) located underneath the first one; (7) placing the collected mites on absorbent paper; (8) counting the collected mites and bees; and (9) determination of the infestation rate as the number of mites found in a sample of bees, divided by the number of bees and multiplied by 100.

Based on the results of the examination with the flotation method, the colonies were divided into three groups A, B and C. Groups A, B and C included colonies featuring the *V.d.* infestation rates given in brackets:

- Group A: A1 (0.6%), A2 (0.3%), A3 (0.2%), A4 (0.0%) and A5 (0.0%);
- Group B: B1 (4.9%), B2 (4.7%), B3 (4.4%), B4 (4.3%) and B5 (3.8%);
- Group C: C1 (52.0%), C2 (30.3%), C3 (11.5%), C4 (13.0%) and C5 (11.0%).

The determination of the *V.d.* infestation rate using a flotation method was done once in the entire period when the gas sensor measurements of the bee colonies were conducted. The obtained results were used as the point of reference in the development of the method based on gas sensing.

### 2.2. Gas Sensor Device

**Figure 1:**
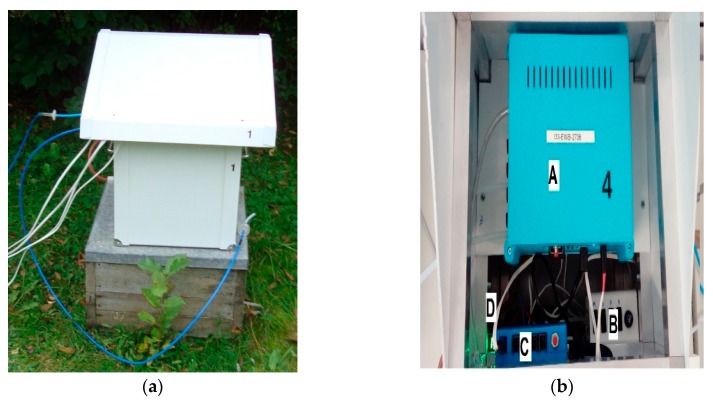
The prototype multisensor detector of air quality, which was applied for beehive air measurements. (**a**) General view and (**b**) top view, disclosing the main functional units: A—the multichannel recorder of gas sensor signals; the gas sensor chamber is mounted inside the recorder; the gas inlets are visible on the left side of the recorder; and the battery was located below the recorder; B—the charging regulator for the solar panel; C—the communication controller, Beecom; and D—the battery level indicator.

The prototype multisensor detector of air quality was applied for the measurements of beehive air, see Figure 1. The device allowed for the continuous exposure of the gas sensors to the gases of interest as well as the sensors’ response measurement and recording. The detailed characteristics of the device were presented in [38]. Further, we elaborate on the selected important aspects of the detector.

The instrument was based on commercially available semiconductor gas sensors, offered by Figaro Engineering Japan [54]. The commercial sensors were used to assure the reliability of the sensing elements. Semiconductor gas sensors were chosen because they fit the requirements of the E-nose technology. This idea was critical for our conception of the detection of bee colony disease, based on beehive air measurements. The device was fitted with the following six semiconductor gas sensors: TGS823, TGS826, TGS832, TGS2600, TGS2602 and TGS2603; see Table 1. They were selected based on the product information sheets [54] and our earlier measurement experiences. The individual sensors (1) exhibit the dominating partial selectivity to different compounds, (2) have different detection ranges and (3) represent different technologies of sensor manufacturing: ceramic (TGS8xx) and thick film technology (TGS2xxx); as shown in Table 1.

The sensors were chosen in view of assembling a gas sensor array that would well reflect the variability of the beehive atmosphere at a relatively low cost. As the beehive air composition is virtually unknown, it was requested that the sensors (1) respond to wide range of volatile compounds, in particular volatile organic compounds; (2) respond in a broad concentration range; and (3) offer insight into the trade-off between the sensing performance and energy consumption of the gas sensor device. The energy consumption of sensors is dependent on the manufacturing technology; however, there are other, more extensive consequences for sensor measurement characteristics.

The applied prototype device operated in the dynamic mode. Namely, the investigated gas was passed through the sensor chamber at a predefined flow rate, for a predefined time. In that period, we recorded responses of gas sensors, which included the information about the measured gas. The information refers to the qualitative as well as quantitative composition. The dynamic mode of operation allowed for the periodic regeneration of gas sensors in the stream of clean air. The important effect of this operation is the baseline restoration. The systematic regeneration also allowed us to reduce the effect of gas sensors drift. Thanks to the dynamic mode of operation the instrument could be designed in a way that allowed for multiple point measurements. Namely, the measured gas may be delivered to the device from multiple sampling points intermittently, while the location of the device is not changed. The applied prototype is fitted with eight gas inlets, so the device may simultaneously monitor seven bee colonies.

The measurement data was collected with a resolution of 1 s, which is sufficient to reflect the momentary temporal changes of beehive atmosphere in gas sensor responses. The data may be recorded locally, on the SD card. The device also has the option of remote data transmission. It is equipped with a GSM module, which allows for a mobile Internet connection using GPRS/EDGE/UMTS/HSDPA/HSUPA/HSPA+ networks. This solution guarantees time synchronization and data backup for offline processing.

The instrument is programmable and automatic. Once programmed and connected to the investigated objects, its operation is virtually maintenance free.

The device is portable. It is prepared for continuous operation in field conditions (e.g., an apiary), including bad weather and a lack of mains. It is fitted with batteries and a photovoltaic solar panel, allowing for 24 h of continuous operation.

### 2.3. Gas Sensor Measurements of Beehive Air

The experimental part of the study consisted of the measurements of honey bee colonies, using the device based on gas sensors, see Figure 1. The measurement session was carried out in field conditions, in September 2019. Fifteen bee colonies were examined, see Section 2.1. The measurements were conducted for five days. Three colonies per day were monitored. 

The beehive air was transported from the sampling points to the gas sensor device using polyethylene tubing, see Figure 1. The gas sampling points were located inside beehives, in the vicinity of the honeycombs with brood. This location was chosen because the *V.d.* infestation rate assessment using the flotation method was based on samples of bees collected from this site as well. The sampling points were accessed from the top of the beehives. The inlet of the tubing, inside the beehive, was protected against clogging by bees with a highly permeable shield. Outside the beehive, the tubing was laid with a tilt. The inlet to the gas sensor device was located above the lowest point of the tubing to prevent instrument damage in the case of water vapor condensation inside the tubing. The inlet of the gas sensor device was protected by the syringe filter against the aerosol, which could be transported from the beehive to the gas sensor chamber. This aerosol interaction with sensors could impair the measurements. One, dedicated gas inlet of the gas sensor device was connected with the ambient air, via a charcoal filter. The filter was used to clean the ambient air and provide regeneration air, which was a reference for the beehive air measurements.

The measurement protocol for an individual beehive was based on several assumptions.

- A sampling point in the beehive was monitored for 24 h.
- In this period, the sampling point was served three times, once every 8 h, approximately; if more measurements were done, the collected data was included in the data analysis.
- Each time the sampling point was served, four single measurements were done, in sequence, one after another.
- A single measurement consisted of two phases (1) the exposure of the gas sensors to beehive air (600 s) and (2) the exposure of the gas sensors to the regeneration air (600 s).

**Figure 2:**
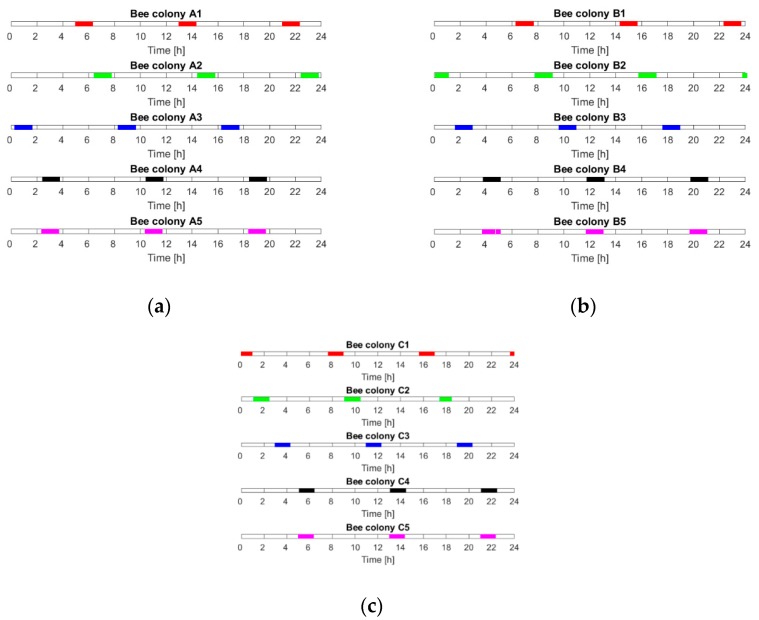
The temporal distribution, within 24 h, of the gas sensor measurements of the beehive air for colonies representing (**a**) Group A, (**b**) Group B and (**c**) Group C.

Figure 2 presents the temporal distribution of the gas sensor measurements of the individual bee colonies during 24 h. The following colonies were monitored on the same day:

- Day 1: A1, B1 and C1;
- Day 2: A2, B2 and C2;
- Day 3: A3, B3 and C3;
- Day 4: A4, B4 and C4;
- Day 5: A5, B5 and C5.

In the 24 h period, the gas sensor device was physically connected to three beehives using three separate gas inlets.

As indicated by the measurement protocol and as shown in Figure 2, the periods of gas sensor measurements of a single beehive were almost evenly distributed around 24 h. However, the measurements of the individual colonies were done at different times of day. As a result of this shift, the entire 24 h-long period was covered by the measurements of various bee colonies. In particular, the sufficient time coverage was attained for the individual groups of bee colonies: for A, see Figure 2a; B, see Figure 2b; and C, see Figure 2c. This property of the measurement data allowed us to examine the temporal (diurnal) aspect of the effectiveness of varroosis detection. In other words, we could determine the performance of varroosis detection based on the measurement data collected at different times of day.

### 2.4. Categories of V.d. Infestation

Based on the feedback from professional beekeepers, we distinguished three levels of bee colonies infestation with *V.d.* mites. These levels are presented in Table 2. 

The infestation levels were defined using the ranges of infestation rates, as detected by a flotation method. The individual levels represented different advancement of the varroosis. Please bear in mind, the level “low” included colonies that had a 0% infestation rate. These could be colonies that were not infested or the colonies for which the infestation rate was below the detection limit of the flotation method.

In this work, the problem of infestation classification was examined Three classification problems were formulated. Their list is presented in Table 3.

Problem P1 consisted of the recognition of the bee colonies that belonged to the “Low” infestation category from all other colonies. Problem P2 was recognition of the bee colonies that belonged to the “Medium” infestation category from all other colonies. Problem P3 was the recognition of the bee colonies that belonged to the “High” infestation category from all other colonies.

### 2.5. Classification and its Performance Assessment

It was assumed that the *V.d.* infestation category of a bee colony could be determined based on a single measurement (see Section 2.3) of the beehive air, using the measurement device based on a gas sensor array.

The gas sensor signals, recorded during a single measurement, were utilized for feature vector construction. In this study, the feature was defined as the value of the sensor signal associated with the single, defined time point of the sensor exposure to the beehive air. This kind of feature is accessible without the additional transformations of the gas sensor signal, except for baseline correction. The simplicity of the feature makes it attractive to avoid the unnecessary complexity of the data processing module embedded in the measurement device. The feature vector was formed by applying the feature selection procedure. The vector included responses collected during the initial 3 min of gas sensor exposure to the beehive air, except for the first 30 s when the sensor signal exhibited a rapid change. The responses of the all sensors, elements of the gas sensor array, were utilized. The employed approach to feature vector formation was based on the results of earlier works on gas sensor measurements using the dynamic mode of operation [37,38,39,55]. In particular, it was demonstrated by Szczurek et al. [37,38,39] that the first several minutes of gas sensor exposure to beehive air were sufficient to attain the effective classification of *V.d.* infestation of a bee colony. 

The feature vector composed of the selected time point responses of gas sensors was used as the input for the classifier, i.e., the basis of the classification. 

Support vector machine (SVM) was applied as a classifier [56]. This kind of classifier is dedicated to binary classification problems and showed high performance in this context. The classifiers were built and tested in the MathWorks environment. 

Separate classifiers were prepared for the individual classification problems P1, P2 and P3. 

**Figure 3:**
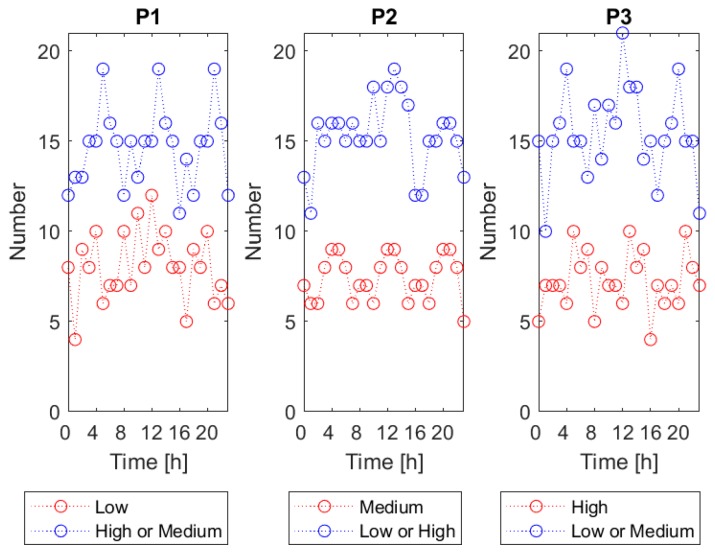
The number of measurement data vectors collected at 3 h-long time intervals, with their centers at 0:00, 1:00, 2:00, … and 23:00. The number of data vectors representing various *V.d.* infestation categories with reference to classification problems P1, P2 and P3 are shown.

The dedicated classification performance assessment procedure was designed. It allowed to examine the recognition performance of the *V.d.* infestation category as a function of the time of day when the gas sensor array measurement was done. The procedure was based on “ten-folds” cross validation. This validation approach consists of running the classification process ten times on one data set. Each time, 90% of the data set is used for training and another 10% is used for testing. Each run, the 10% used for testing comprises different subset of the entire data set, which is evenly distributed among the classes. As a result, after completing the “ten-folds” cross-validation the predicted class assignment is known for all input data vectors, in conditions when they were excluded from the training set. For examining the classification performance, we grouped the input data vectors based on the time of measurement. For that purpose, the 24 h period was divided into 3 h-long time intervals. The centers of the successive intervals were: 0:00, 1:00, 2:00, … and 23:00. The number of data vectors included in the successive time intervals is shown in Figure 3, with reference to the classification problems P1, P2 and P3. On average, in a single time interval, the bigger category was represented by fifteen data vectors and the smaller by seven to eight data vectors. The minimum number of data vectors, in one time interval, was four. 

The classification performance was assessed in the individual time intervals, based on the results of the classification of the data vectors, which belonged to these intervals. Additionally, the average performance was assessed, for the entire 24 h-long period of time, based on all the data vectors. The confusion matrix, see Table 4, was applied to assess the performance of the *V.d.* infestation assessment based on the classification.

Depending on the examined classification problem, the categories quoted in Table 4 shall be referred as follows:

- Problem P1: Category 1—“Low”, Category 2—“High or Medium”;
- Problem P2: Category 1—“Medium”, Category 2—“Low or High”;
- Problem P3: Category 1—“High”, Category 2—“Low or Medium”.

The following performance indicators were derived based on the confusion matrix (see Table 4): the true positive rate (TPR = n_11_/(n_11_ + n_12_)), true negative rate (TNR = TPR = n_22_/(n_21_ + n_22_)) and the average of the two ((TPR + TNR)/2).

## 3. Results

### 3.1. The Diurnal Variation of Gas Sensor Responses to Beehive Air

**Figure 4:**
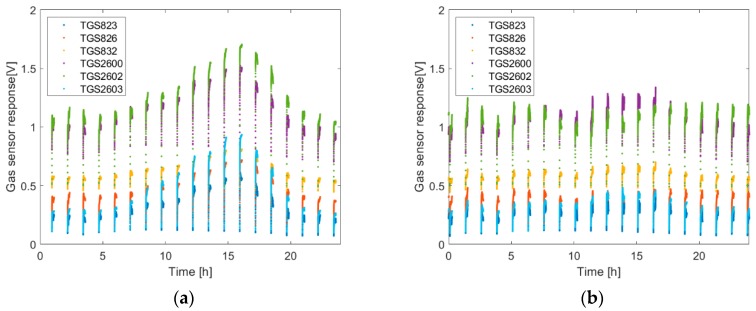
The temporal variation, within 24 h, of the gas sensor responses to beehive air for two bee colonies: (**a**) Bee Colony 1 and (**b**) Bee Colony 2.

Figure 4 shows the exemplary results of the gas sensor measurements of the beehive air over the course of 24 h. The measurements were done for two bee colonies on the same day, in May 2019.

As shown in Figure 4, the responses of the semiconductor gas sensors to the beehive air displayed diurnal variations. However, the magnitude of the responses, as well as the character of their changes, could be different depending on the beehive, even on the same day.

### 3.2. Performance of the V.d. Infestation Category Detection vs. the Time of Day

Table 5 presents the average TPR, TNR and (TPR + TNR)/2 for the detection of the *V.d.* infestation categories, with the measurement data collected during 3 h-long time intervals, evenly distributed within 24 h. The standard deviation for these indicators is shown in Table 6, respectively.

Based on results shown in Table 5, we observed a consistent disproportion between the true negative rates and true positive rates, irrespective of the infestation category. The true negative rates were high. In all cases, they exceeded TNR = 0.9000. The values of the indicator were similar for all infestation categories, i.e., from TNR = 0.926 (category “Low”) to TNR = 0.969 (category “High”). The true positive rates were lower, with a minimum value of TPR = 0.656 (category “Medium”). This indicator varied highly among the categories of *V.d.* infestation. The highest value, TPR = 0.754, was associated with the detection of the infestation category “Low”, and it was similar in case of the category “High”, with a TPR = 0.743. The disproportion between the TPR and TNR indicates that the limiting factor for the successful classification was the correct recognition of colonies that truly belonged to Category 1, see Section 2.5 (as shown by TPR), as the other colonies were generally successfully identified as not belonging to this category (as indicated by TNR).

As shown in Table 6, the magnitude of the variation of the TPRs, in the period of 24 h, was very different as compared with the TNRs. For all categories of infestation, the 3 h-TPRs displayed a substantial temporal variation. This was the highest in the case of the category “Medium”, when the std (TPR) = 0.268 indicated a 40% variation around the mean daily value of the indicator. For the infestation category “Low” the daily spread of the 3 h-TPR constituted 31% of the mean level of the indicator. For the category “High”, the TPR spread was 30% of the mean daily value. The substantial changes of the TPR in the period of 24 h imply the importance of the time of day when the measurement was done for the effective recognition of the *V.d.* infestation category of the bee colony. Compared with the TPR variability, the TNRs were nearly constant during the day, except for the category “Low”; in this case, the spread, with a std (TPR) = 0.0995, was about 11% of the daily average value of the TPR.

**Figure 5:**
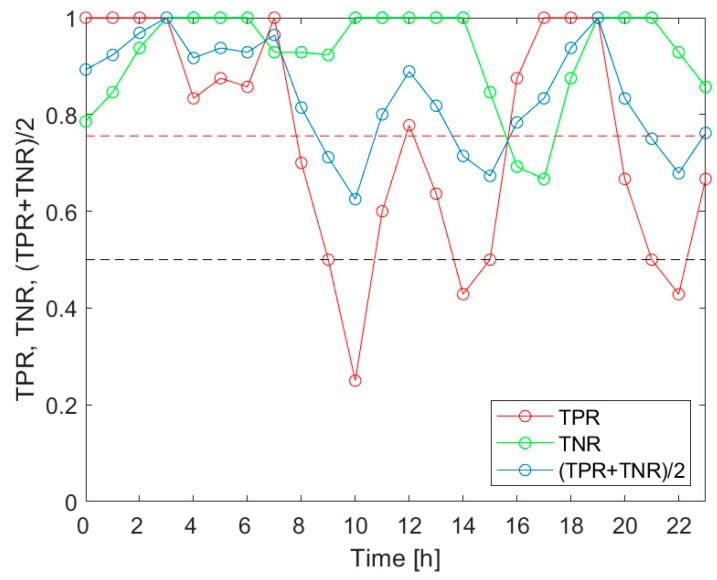
The detection performance indicators of the *V.d.* infestation category “Low”, for the measurement data collected during 3 h-long time intervals, evenly distributed within 24 h. The black dashed line indicates the cut-off level for all performance indicators, i.e., 0.5. The red dashed line shows the average value of the TPR.

**Figure 6:**
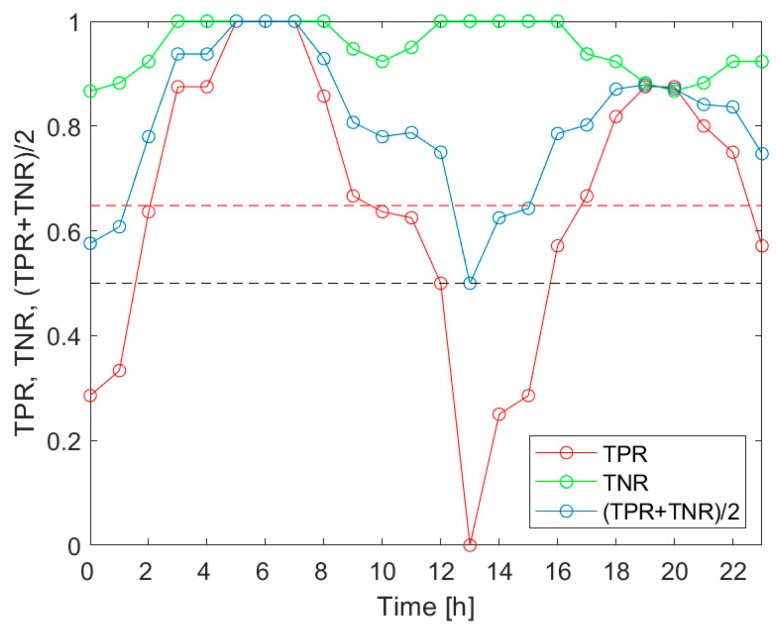
The detection performance indicators of the *V.d.* infestation category “Medium”, for the measurement data collected during 3 h-long time intervals, evenly distributed within 24 h. The black dashed line indicates the cut-off level for all performance indicators, i.e., 0.5. The red dashed line shows the average value of the TPR.

**Figure 7:**
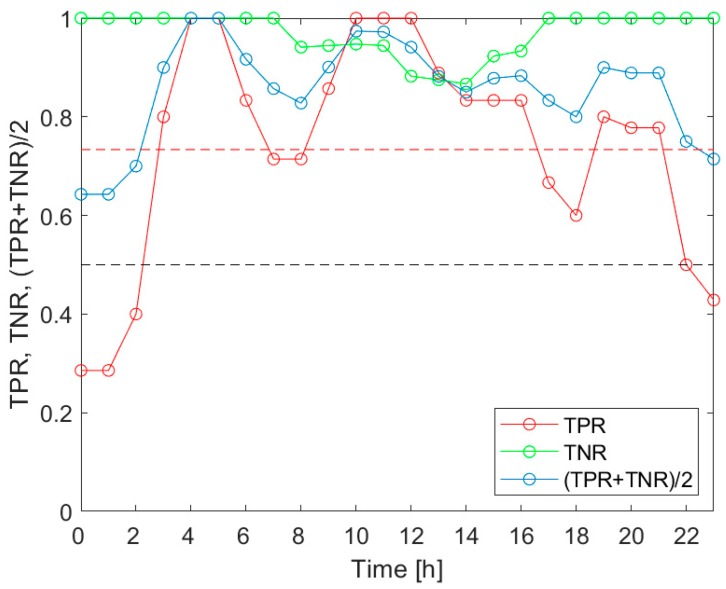
The detection performance indicators of the *V.d.* infestation category “High”, for the measurement data collected during 3 h-long time intervals, evenly distributed within 24 h. The black dashed line indicates the cut-off level for all performance indicators, i.e., 0.5. The red dashed line shows the average value of the TPR.

Figure 5 presents the TPR, TNR and (TPR+TNR)/2 associated with the detection of the *V.d.* infestation category “Low”, for the measurement data collected during the 3 h-long time intervals, evenly distributed within 24 h. The indicators of the classification performance are shown as a function of the time of day. The markers in the plots indicate the middle points of the respective 3 h time intervals, which provided the measurement data. Figure 6 displays the results related to the detection of the *V.d.* infestation category “Medium”, and Figure 7 refers to the category “High”.

As shown in Figure 5, Figure 6 and Figure 7, the indicators of the effectiveness of the *V.d.* infestation classification, TPR, TNR and (TPR+TNR)/2, displayed the variation in the period of 24 h. In particular, the most altered parameter was the true positive rate of detection. Based on the comparison of Figure 5, Figure 6 and Figure 7, the diurnal variation of the performance indicators was dependent on the detected categories of the *V.d.* infestation. These facts imply that (1) the performance of the *V.d.* infestation classification was highly dependent on the time of day when the measurement was done; and (2) the detection of different categories of infestation could not be equally effective, when based on the measurement data collected at different times of day.

Based on Figure 5, the *V.d.* infestation category “Low” was detected more effectively than on average, when the beehive air measurements were performed between 23:00 and 8:00 a.m., between 11:00 and 13:00 and between 15:00 and 20:00. This category was best detected between midnight and early morning, late afternoon and early evening; see Figure 5. In the case of the *V.d.* infestation category “Medium”, the 3 h-TPRs exceeded the 24 h average TPR between 2:00 and 10:00 a.m., as well as between 16:00 and 22:00; see Figure 6. This category was most effectively detected in the morning. Considering the detection of the *V.d.* infestation category “High”, the 3 h-TPRs were greater than the 24 h average TPR between 2:00 and 7:00 a.m., from 8:00 to 17:00 and between 18:00 and 22:00; see Figure 7. For this category, the preferred time of detection was early morning and midday.

In Table 7, we summarized the times of day, when the TPR for the classification of the data collected in 3 h-long intervals exceeded the average for the 24 h period. The most favorable times of day were compared, related to the detection of the *V.d.* infestation categories “Low”, “Medium” and “High”. The common parts of the intervals were marked in grey. Table 7 shows two periods during the 24 h when the detection of all infestation categories was possible with the performance greater than the 24 h average. They were the 7 h-long period between 1:30 a.m. and 7:30 a.m. and the 3 h-long period between 17:30 and 20:30. Based on this summary, the favorable period for gas sensor measurements aimed at *V.d.* infestation assessment of the bee colony was very early morning. 

## 4. Discussion

The objective of this work was to examine the effectiveness of the *V.d.* infestation classification as a function of the time of day when the gas sensor measurements were performed. The consideration of this problem was justified by the observation that semiconductor gas sensor responses change during the day while the infestation rate of the bee colony remains constant in this period of time.

Several researchers observed temporal changes in beehive air parameters, including concentrations of selected gases. Due to methodological constraints, not many gases were measured with a temporal resolution that is sufficient to observe the diurnal variation of their concentrations. Meikle and Holst [14] quoted the results obtained by other researchers before 2015. Among them, Human et al. [41] showed the variation in microclimatic parameters in three individual bee colonies over four consecutive days at 2-min intervals. The examined parameters were temperature, absolute humidity and relative humidity. Researchers detected a circadian rhythm of these parameters. The changes in beehive air humidity were explained by bee colony activity, in particular, the active concentration of nectar by tongue lashing [42], which begins just after unloading [43] and stops when foraging is completed, at dusk. Seeley [44] indicated that humidity can also depend on trade-offs with other biophysical parameters, such as temperature or respiratory gases, for example. Additionally, it is influenced by external factors, such as water or nectar availability outside the hive. The daily fluctuations of humidity in the brood area and nectar stores could be due to the honey ripening process.

Fanning behavior of bees, which is an effective mechanism of beehive ventilation, was discussed by Southwick and Moritz [45]. It has a form of a breathing pattern. As a result, the beehive atmosphere is intermittently diluted and concentrated. The colonial respiratory activity follows a pronounced day–night cycle and it is decreased at night. According to Seeley [44] the fanning behavior in the honey-bee is driven by the concentration of CO_2_. The concentration of this respiratory gas may be regulated in the beehive atmosphere by 0.10% and 4.25% in small colonies, while large ones are capable of more precise control. Ohashi et al. [46] found that the hive CO_2_ concentration fluctuated corresponding to the hive temperature even when the atmospheric CO_2_ concentration was stable. Ohashi et al. [47] reported that sometimes they also observed a drastic increase in CO_2_ concentration at midday, which might be caused by the circadian rhythm of the honey bees [48]. Ohashi et al. [46] found a significant increase in the CO_2_ concentration 6 a.m. each day. Another CO_2_ peak was observed in the early evening.

Even though the temperature, relative humidity and CO_2_ concentration in the beehive were monitored by relatively many researchers, they have not identified a unique, daily pattern of their variability. Contrarily, various kinds of variation were observed. Researchers keep on formulating hypothesis concerning the reasons why the listed parameters fluctuate in the beehive, differently from those outside, and how honey bees control these factors purposely. Yet they claim it is still unclear and more work has to be done [46,49].

The published results of investigation of beehive microclimate and its regulation mechanism entitle us to claim that the concentration of volatile compounds in the beehive atmosphere is also not constant in time. It should rather be expected that the concentrations of these gases change. The relevant published results are scare. Edwards-Murphy et al. [50] reported the pattern observed in the pollutant sensors (“nitrogen dioxide, and raw pollutant sensor data”). It consisted of sensor responses increasing over time until weathering by beehive roof opening. After that, the signals decreased. The recorded responses were interpreted as caused by pollutant build-up in beehive air, followed by their removal.

Based on our measurements, we observed the diurnal changes of the semiconductor gas sensor responses to beehive air. The character of daily variation could be different. In our opinion, this is a result of multiple factors influencing the composition of beehive air. For sure, the mechanisms responsible for thermal regulation as well as humidity and CO_2_ concentration control are involved. We think that also foraging activity plays an important role. It is related to the variation of beehive occupancy [51], the composition of the collected pollen mixture [52] and concentration of nectar [42]. However, the distinguishing of categories of diurnal changes, and further, identification of factors which are responsible for the particular kinds of variation, requires substantial further research.

No justified comments can be made, at this point, regarding the daily variation of the gas sensor responses with respect to *V.d.* infestation of bee colonies. In principle, the infestation is a progressive process. No published information was found about the time of mite’s developmental stages being equal to 24 h. Therefore, based on the current knowledge, the diurnal variation of the beehive atmosphere shall be considered as an interfering factor from the point of view of determining the *V.d.* infestation of a bee colony, based on gas sensor measurements. This is the fact that underlines the importance of our study.

The presented *V.d.* infestation detection method was based on the measurements of beehive air using semiconductor gas sensors. Technically, a single measurement can be realized at any time of day. However, based on the results presented in Figure 4, one may expect different measurement results of the same beehive depending on the time of day. This raises the issue of the general character of the transformation between the pattern of beehive air contained in gas sensor array responses, which changes during the day, and the infestation rate of bee colony, which is assumed constant during the day, even during several days. The issue may be also stated differently, as a question about the common part of the information contained in all the measurement data, irrespective of the time of measurement. This information shall be relevant and represent the degree of the bee colony infestation by the mite. 

The working hypothesis of this work was that, in the measurement data collected in a period of 24 h, there is a certain level of common information, which is relevant for the *V.d.* infestation assessment. Based on the experimental data, we demonstrated that there were times of day when the measurement data was more relevant, and therefore the *V.d.* infestation determination was highly efficient. There also existed other times of day, when the relevance of the information in the gas sensor responses decreased, potentially even eliminating the possibility of successful detection of the disease.

Our investigation confirmed the working hypothesis. As shown in Figure 5, Figure 6 and Figure 7, the true positive rates of the *V.d.* infestation classification varied considerably within 24 h. The TPR was the actual probe of the detection performance, as the true negative rate was high and relatively invariant during the day. In the course of the analysis, we identified the times of day when the infestation classification was very effective. We also indicated the periods of day when the detection of the disease was completely unsuccessful. These were situations when at least one of the classification performance indicators was smaller than 0.5. Moreover, the obtained results showed that the temporal variation of the classification performance was dependent on the category of infestation. This obtained result is very intriguing. Unfortunately, at this stage of our research, we are not able to elaborate on the observed differences in *V.d.* detection performance with reference to the bee colony biology.

The performed analysis was based on a relatively small sample of fifteen bee colonies. Although the experiment was performed under strict rigor, on warm September days, the presented results shall be considered as the indication of a problem that needs to be carefully addressed. The identification of early morning hours as the time of day when the detection of all categories of *V.d.* infestation was highly effective should not be taken as the ground truth. It is possible that under different conditions, e.g., meteorological ones, the results could be different. More experiments are needed to determine and recommend, in a responsible manner, the specific times of the day as consistently favorable for performing measurements for effective *V.d.* infestation classification. The results presented in this paper could help in planning the respective studies.

The problem addressed in this work is very important from the point of view of beekeeping practice and the application of sensor technology in the precision agriculture. The obtained results draw attention to the potential necessity of imposing restrictions—that certain periods of day are recommended for performing measurements and other periods are prohibitive in that respect. This information would be very important for beekeepers, who have to plan the measurement activities in the apiary. 

## 5. Conclusions

This work examined the problem of *V.d.* infestation classification based on beehive air measurements, which were performed at different times of day. The motivation for the study was the experimentally confirmed diurnal variation of semiconductor gas sensor responses to beehive air.

We considered three categories of infestation: “Low”, “Medium” and “High”. Each category was detected individually, in the framework of the respectively formulated binary classification problem.

The performance of the detection was evaluated using the true positive rate and true negative rate. These indicators were calculated using the results of the classification for the data associated with the 3 h-long intervals of measurements, which were evenly distributed over the 24 h.

Based on the analysis, the performance of the detection varied over the 24 h period. The most favorable times of day were different depending on the category of infestation. The identified common parts pointed at the very early morning as the most favorable period for gas sensor measurements aimed at *V.d.* infestation assessment of a bee colony.

The proposed measurement method is at the early stage of development. We already know that many factors have an influence on its performance. Our future work will concentrate on the better understanding of these factors. We expect that this may result in the modification of the sampling as well as the measurement procedure.

## Figures and Tables

**Table 1:** The sensitivity and the detection ranges of the applied sensors [54].

Gas Sensor	Sensitivity	Detection Range
TGS 823	Solvent vapors	50–5000 ppm Ethanol, n-Hexane, Benzene, Acetone
TGS 826	Ammonia	30–300 ppm Ethanol, Ammonia, Isobutane
TGS 832	Chlorofluorocarbons	10–600 ppm ethanol, R-407c, R-134a, R-410a, R-404a, R-22
TGS 2600	Air contaminants	1–100 ppm Ethanol, Isobutane, Hydrogen, Carbon monoxide
TGS 2602	Volatile organic compounds and odorous gases	1–30 ppm Ethanol, Ammonia, Toluene
TGS 2603	Amine series and sulfurous odor gases	1–30 ppm Ethanol 0.1–3 ppm Trimethyl amine, 0.3–2 ppm Methyl mercaptan

**Table 2:** The categorization of honey bee colony infestation by *V.d*.

Level of Infestation	*V.d*. Infestation Rate of Bee Colonies (%)	Group of Experimental Bee Colonies ^1^
Low	0–2	A
Medium	2–6	B
High	>6	C

^1^ See Section 2.1 for the details of infestation rates of the individual bee colonies in Groups A, B and C.

**Table 3:** Classification problems.

Classification Problem	Category of Infestation	*V.d*. Infestation Rate of Bee Colonies (%)	Group of Experimental Bee Colonies ^1^
P1	“Low”	0–2	A
	“Medium or High”	>2	B, C
P2	“Medium”	2–6	B
	“Low or High”	0–2 or >6	A, C
P3	“High”	>6	C
	“Low or Medium”	0–6	A, B

^1^ See Section 2.1 for the details of infestation rates of the individual bee colonies in Groups A, B and C.

**Table 4:** The generic confusion matrix for the classification problems considered in this work.

	Category 1—Predicted	Category 2—Predicted
Category 1—true	n_11_	n_12_
Category 2—true	n_21_	n_22_

**Table 5:** The average of the TPR, TNR and (TPR + TNR)/2 associated with the detection of the *V.d.* infestation categories, for the measurement data collected during 3 h-long time intervals, evenly distributed within 24 h.

Classification Problem	TPR ^1^	TNR ^2^	(TPR + TNR)/2
P1	7.54 × 10^−1^	9.26 × 10^−1^	8.40 × 10^−1^
P2	6.56 × 10^−1^	9.51 × 10^−1^	8.04 × 10^−1^
P3	7.43 × 10^−1^	9.69 × 10^−1^	8.56 × 10^−1^

^1.^ True positive rate, see Section 2.5; ^2.^ True negative rate, see Section 2.5.

**Table 6:** The standard deviation of the TPR, TNR and (TPR + TNR)/2 associated with the detection during 3 h-long time intervals, evenly distributed within 24 h.

Classification Problem	TPR ^1^	TNR ^2^	(TPR + TNR)/2
P1	2.33 × 10^−1^	9.95 × 10^−2^	1.10 × 10^−1^
P2	2.68 × 10^−1^	5.06 × 10^−2^	1.37 × 10^−1^
P3	2.21 × 10^−1^	4.52 × 10^−2^	1.02 × 10^−1^

^1.^ True positive rate, see Section 2.5; ^2.^ True negative rate, see Section 2.5.

**Table 7:** The time of day when the TPR for the classification of the data collected in 3 h-long time intervals exceeded the average TPR 24 h period. The times of day indicate the middle points of the individual 3 h-long time intervals.

Infestation Category	Time of Day (h)																							
	0	1	2	3	4	5	6	7	8	9	10	11	12	13	14	15	16	17	18	19	20	21	22	23
Low	x	x	x	x	x	x	x	x					x				x	x	x	x				
Medium				x	x	x	x	x	x	x								x	x	x	x	x		
High				x	x	x	x			x	x	x	x	x	x	x	x			x	x	x		
